# Utrecht Gender Dysphoria Scale – Gender Spectrum in a Chinese population: scale validation and associations with mental health, self-harm and suicidality

**DOI:** 10.1192/bjo.2022.617

**Published:** 2023-01-18

**Authors:** Runsen Chen, Yi Feng, Di Su, Amanda Wilson, Meng Han, Yuanyuan Wang

**Affiliations:** Vanke School of Public Health, Tsinghua University, China; and Institute for Healthy China, Tsinghua University, China; Mental Health Center, Central University of Finance and Economics, China; and Faculty of Psychology, Beijing Normal University, China; Department of Psychology, Tsinghua University, Beijing, China; and Mental Health Center, Ningxia University, China; Division of Psychology, Faculty of Health and Life Sciences, De Montfort University, UK; Vanke School of Public Health, Tsinghua University, China; Key Laboratory of Brain, Cognition and Education Sciences, Ministry of Education, China; School of Psychology, South China Normal University, China; Center for Studies of Psychological Application, South China Normal University, China; and Guangdong Key Laboratory of Mental Health and Cognitive Science, South China Normal University, China

**Keywords:** Depression, anxiety, gender dysphoria, suicide, psychological psychometrics

## Abstract

**Background:**

Individuals with gender dysphoria display an incongruence between birth-assigned gender and gender expression. However, there is no existing Chinese measure for gender dysphoria.

**Aims:**

This study aims to validate the Utrecht Gender Dysphoria Scale – Gender Spectrum (UGDS-GS) in a Chinese population, and compare the psychometric properties of the UGDS-GS with one frequently used scale for gender dysphoria measurement, the Gender Identity/Gender Dysphoria Questionnaire for Adolescents and Adults (GIDYQ-AA).

**Method:**

A total of 2646 Chinese participants were recruited. The following information was collected: sociodemographic variables, gender identity, sexual orientation, gender dysphoria measured by the UGDS-GS and the GIDYQ-AA, anxiety, depression and suicide assessment. Principal component analyses and confirmatory factor analysis (CFA) were conducted to test the fitness of the model. Discriminant validity was tested with one-way analysis of variance.

**Results:**

The UGDS-GS showed good psychometric properties, with the GIDYQ-AA demonstrating slightly better psychometric properties than the UGDS-GS. UGDS-GS also showed strong internal consistency (Cronbach's *α* = 0.89), and good convergent validity and criterion validity. Exploratory factor analysis showed a one-factor structure (Kaiser-Meyer-Olkin test, 0.93; *χ^2^* = 13 342.50; d.f. = 153; *P* < 0.001). The UGDS-GS was positively associated with anxiety symptoms, depressive symptoms, suicidal ideation, attempted suicide and self-harm. We also found the results were robust in different samples.

**Conclusions:**

The validated UGDS-GS can significantly stimulate and promote gender dysphoria assessment in Chinese populations, allowing for assessment in a more diverse subset of gender minorities.

Gender dysphoria has been a central focus in transgender healthcare.^1^ It is well-documented that individuals with gender dysphoria experience distress from multiple avenues, including in their personal, social and occupational lives.^2^ The DSM-5^3^ defines gender dysphoria as an marked incongruence between an individual's experienced gender and birth-assigned gender. However, not all people who experience gender dysphoria meet the DSM-5 gender dysphoria diagnosis because not all individuals seek gender-affirmative treatment.^4^ In addition, the standardised diagnostic instrument Structured Clinical Interview for the DSM requires trained clinical professionals and is time-consuming,^5^ which is difficult to apply efficiently in the general population. To achieve an in-depth understanding of gender dysphoria and promote the health of individuals with gender dysphoria, it is important to provide valid and reliable gender dysphoria assessment, outside of the DSM diagnosis criteria. The Utrecht Gender Dysphoria Scale (UGDS)^6^ and the Gender Identity/Gender Dysphoria Questionnaire for Adolescents and Adults (GIDYQ-AA)^7^ are the two most widely used scales to assess for gender dysphoria, using two versions of the measures, one male and one female, which are based on birth-assigned gender.

## Gender dysphoria assessment

The UGDS is a 12-item screening measure for gender dysphoria in both adults and adolescents.^6^ The GIDYQ-AA is a 27-item scale for gender identity and gender dysphoria in both adolescents and adults.^7^ Both the UGDS^4,8,9^ and GIDYQ-AA^10–13^ have been validated and widely applied in various settings, with different age groups. Furthermore, those two scales are significantly correlated with each other.^14,15^

Recent research has moved beyond focusing on assigned gender and binary conceptualisation of transgender identity, to be inclusive of non-binary transgender identities.^12,16,17^ However, non-binary people may feel uncomfortable responding to either a male or female version of the gender dysphoria scales based on their fluid identity.^18^ In addition, researchers have noted that gender dysphoria scales with distinct male and female versions, such as the UGDS, are less than ideal for detecting gender dysphoria in a genderqueer or genderfluid individual.^4^ Moreover, to support people with disorders of sex development (DSDs)/intersex conditions, instruments are required to specifically measure gender dysphoria taking non-binary gender identity into account.^8^ The DSM-5 defined DSDs as a specifier of gender dysphoria, that is, gender dysphoria with or without a DSD.^3^ Researchers have commented that this change in the DSM-5 was unprecedented and saw DSDs subsumed under psychiatric disorders, with an emphasis placed on the psychiatric conditions of people with DSDs.^19^ It is therefore necessary that suitable psychiatric and mental health measurements for people with DSDs, especially for gender dysphoria, are validated.

The Utrecht Gender Dysphoria Scale – Gender Spectrum (UGDS-GS) is an adapted version of the original UGDS, which combines both versions of the UGDS to create a 18-item gender-neutral measurement assessing gender dysphoria on a continuum spectrum.^18^ The UGDS-GS reconstructed the original UGDS to provide more fluid movement along the gender spectrum, making it suitable to measure for gender dysphoria in non-binary individuals, individuals undergoing gender affirmation surgery and people with DSDs. The UGDS-GS is a newly developed scale, which has yet to be validated in other countries/languages.

This study aimed to validate the UGDS-GS in a Chinese population, and examined the applicability of the UGDS-GS and the GIDYQ-AA for gender dysphoria. The two scales were compared in terms of gender dysphoria conceptualisation, psychometric properties and application in different groups, including transgender, non-binary, genderqueer, cisgender sexual minority and heterosexual individuals. We hypothesised that the Chinese version of the UGDS-GS would demonstrate the same factor structure as the English version, with good psychometric properties. We further hypothesised that the UGDS-GS and GIDYQ-AA would demonstrate different prediction properties in the different groups, especially for non-binary and genderqueer groups, and that the UGDS-GS would outperform the GIDYQ-AA in assessing gender dysphoria in non-binary and queer individuals. Finally, we hypothesised that there would be different predictions in the mental health outcomes of individuals with gender dysphoria.

## Method

### Participants and procedure

This study was conducted from 26 October to 6 November 2020 in the Ningxia Province, China. Adolescent and young adults from local colleges were invited to complete an online survey by distribution of a questionnaire link on the platform ‘Wenjuanxing’, which provides a data collection function. All participants remained anonymous and participants were informed that they could withdraw from the survey at any time before submitting their responses. All participants provided informed consent before they completed the survey, which took on average 10–20 min to complete. A total of 2663 participants completed the survey; 17 samples were excluded owing to incomplete information, leaving 2646 (99.4%) study participants.

The authors assert that all procedures contributing to this work comply with the ethical standards of the relevant national and institutional committees on human experimentation and with the Helsinki Declaration of 1975, as revised in 2008. All procedures involving human patients were approved by Research Ethics Review Committee of Central University of Finance and Economics, China (approval number: CUFE-20200930-0001). Participants’ informed consent was signed online as written consent.

### Measures

#### Sociodemographic characteristics and sexual orientation

Sociodemographic characteristics included age, birth-assigned sex, ethnic group, residence type, family economic status, whether they were the only child in the family, any history of psychiatric disorders and medication status. Sexual orientation was assessed through gender identity and sexual attraction. Gender identity was measured by a single question: ‘Which of the following best describes your gender?’ Responses included six categories: male, female, transgender female, transgender male, non-binary and genderqueer. Sexual attraction was assessed by another question: ‘Which of the following best describes your sexual attraction?’. Responses were classified into five categories: heterosexual, bisexual, homosexual (lesbian/gay), queer and other (e.g. asexual).

#### Gender identity/dysphoria

The UGDS-GS was used to measure the level of gender dysphoria.^18^ It consists of 18 items on a five-point Likert scale ranging from 1 (*‘*disagree completely’) to 5 (‘agree completely’). Example items include ‘I prefer to behave like my affirmed gender’ and ‘Every time someone treats me like my assigned sex, I feel hurt’. All item scores were added to generate a total score, with a higher score indicating a higher degree of gender dysphoria. UGDS-GS is composed of two subscales: a 14-item dysphoria subscale and a four-item gender affirmation subscale. The adaptation of the Chinese version of the UGDS-GS was authorised by the author of the original English version. The process of translation followed the recommended procedures for cross-cultural scale adaptation. Initial translation was conducted by two bilingual native Chinese translators, synthesis of translation by a third bilingual Chinese translator, back translation by two bilingual native English speakers and then an expert review by several psychologists, psychiatrists and medical staff. We also conducted a pre-test with convenience sampling, before testing the final proposed measure.

The GIDYQ-AA^7^ was also used to measure gender dysphoria, to allow for comparison of the two scales on psychometric properties and actual application in Chinese populations. The GIDYQ-AA consists of a male version and a female version, with 27 items for each version. For the male version, it includes items such as ‘In the past 12 months, have you felt satisfied being a man?’ and ‘In the past 12 months, have you disliked your body because it is male (e.g. having a penis or having hair on your chest, arms and legs)?’ For the female version, example items include ‘In the past 12 months, have you felt satisfied being a woman?’ and ‘In the past 12 months, have you disliked your body because it is female (e.g. having breasts or having a vagina)?’ In this study, we reverse-coded the 27 items to a new scoring that ranged from 1 (‘never’) to 5 (‘always’), for easier understanding and statistical comparison with the UGDS-GS. We calculated the total score by adding all item scores together (Cronbach's *α* = 0.90), with a higher score indicating higher gender dysphoria. In our previous study, the recommended cut-off score was 48 for the Chinese version of the GIDYQ-AA.^20^

#### Mental health outcomes

Mental health-related indicators of anxiety symptoms, depressive symptoms, suicidal ideation, attempted suicide and self-harm were measured. Anxiety was measured with the seven-item Generalised Anxiety Disorder Scale (GAD-7), which is a self-report screening scale used to measure anxiety symptoms.^21^ It has been validated in China.^22^ It is composed of seven items, and participants are asked to indicate the frequency of the occurrence of symptoms (e.g. ‘feeling nervous, anxious or on edge’, ‘not being able to stop or control worrying’) over the past 2 weeks on a four-point scale (0 = not at all, 1 = several days, 2 = more than half of the days, 3 = nearly every day). We calculated a composite anxiety score by summing all item scores (Cronbach's *α* = 0.93). Higher scores indicate more severe anxiety symptoms.

The nine-item Patient Health Questionnaire (PHQ-9) was used to assess depressive symptoms.^23^ Similar to the GAD-7, the PHQ-9 has been validated in the Chinese context.^24^ It includes nine self-screening items concerning the frequency of depressive symptoms over the past 2 weeks. For example, ‘little interest or pleasure in doing things’ and ‘thoughts that you would be better off dead or of hurting yourself in some way’. Participants were asked to rate symptoms on a four-point scale, varying from 0 (‘not at all’) to 3 (‘nearly every day’). All items are summed to generate a composite depression score (Cronbach's *α* = 0.92), with higher scores indicating more severe depressive symptoms.

Suicidal ideation was assessed through a single question: ‘How often have you had suicidal thoughts over the past 12 months?’ Participants were asked to respond on a four-point scale (1 = never, 2 = once, 3 = twice, 4 = more than twice). Attempted suicide was measured by a single question: ‘Have you ever attempted suicide?’ The responses was rated on a four-point scale ranging from 1 (‘never’) to 4 (‘more than twice’). Self-harm behaviours was also assessed by a single question (i.e. ‘In the past 12 months, have you ever intentionally harmed yourself without wanting to die?’). Response options were rated on a six-point scale (1 = never, 2 = once, 3 = two to five times, 4 = six to ten times, 5 = 11–20 times, 6 = more than 20 times).

### Analytic approach

#### Validation of the UGDS-GS

All statistical analyses were conducted with the following Windows software: IBM SPSS version 23.0, Mplus version 8.3 (https://www.statmodel.com/) and R version 4.0.2 (https://cran.r-project.org/). Descriptive statistics were generated for each item score and the sociodemographic characteristics. To evaluate the construct validity of the two-factor UGDS-GS in China, we split the sample randomly half by half. An exploratory factor analysis (EFA) including half of the sample was conducted with principal component analyses (PCA) and direct oblimin rotation. A confirmatory factor analysis (CFA) including the other half of the sample was performed by maximum likelihood estimates, to confirm the fitness of the model derived from EFA. The goodness-of-fit model was evaluated by a number of statistics, i.e. *χ^2^/*d.f. ratio, root mean square error of approximation (RMSEA), comparative fit index (CFI), Tucker–Lewis index (TLI) and standardised root mean residual (SRMR).^25^ Acceptable goodness-of-fit model parameters were defined as RMSEA < 0.08, CFI > 0.90, TLI > 0.90 and SRMR < 0.08.^26^

To assess discriminant validity, group difference regarding the total UGDS-GS mean score was compared by a one-way analysis of variance (ANOVA), using the Scheffe's procedure as a *post hoc* test. Cronbach's alphas were calculated to check the reliability of the Chinese version of the UGDS-GS and the two subscales, with *α* = 0.80–0.90 indicting a good fit and *α* > 0.90 indicating excellent internal consistency reliability. An item analysis was also performed to calculate corrected item-total correlation coefficients. To assess the criterion-related validity, Pearson correlations were performed by calculating the correlation between the UGDS-GS score and other mental health variables. Meanwhile, Pearson correlations between UGDS-GS and GIDYQ-AA were calculated to assess the convergent validity of the UGDS-GS. Sensitivity and specificity of the Chinese version of the UGDS-GS were assessed by receiver operating characteristic (ROC) curves. Based on Youden's index, the maximum value of *J* (sensitivity + specificity – 1) was calculated as the optimum cut-off score in the Chinese version.^27^ The statistical significance level was set at two-sided 0.05 *P* value in this study.

#### Comparison of the UGDS-GS and GIDQY-AA

To compare the psychometric properties and application of the UGDS-GS and the GIDYQ-AA in China, we compared the reliability, discriminant validity, criterion-related validity and ROC curves of the two scales. Cronbach's alpha was calculated to assess internal consistency reliability. Cohen's kappa coefficient was calculated to measure interrater reliability between the two scales, with *κ* < 0.40 defined as poor agreement, *κ* = 0.40–0.75 as fair to good agreement and *κ* > 0.75 as excellent agreement.^28^

Discriminant validity of the two scales was compared by performing a one-way ANOVA and paired *t*-test among different gender identity groups and different sexual attraction groups. Criterion validity was compared by Pearson correlations between UGDS-GS, GIDYQ-AA and mental health outcomes. According to Youden's *J*-statistic, the optimistic cut-off scores of both the UGDS-GS and GIDYQ-AA were calculated and corresponding sensitivity, specificity and area under the curve (AUC) were compared between the two scales.

## Results

### Sociodemographic characteristics

A total of 2646 participants constructed the final sample (Table 1). The age ranged from 15 to 28 years (mean 19.30, s.d. = 1.20). The majority of the participants were birth-assigned female (65.6%), ethnic Han (54.7%), urban dwellers (72.8%), with moderate family economic status (66.0%) and they were not the only child in the family (83.4%). There were 4.6% participants who were diagnosed with psychiatric disorders, and 1.6% were on psychiatric medication during the survey.

**Table 1:** Sociodemographic characteristics of participants (*N* = 2646)

Variable	Cisgender	Transgender	Non-binary/genderqueer
		Mean ± s.d./*n* (%)	
Age, years (19.30 ± 1.20)	19.31 ± 1.21	19.13 ± 0.91	18.90 ± 1.05
Birth-assigned gender			
Male (*n* = 910)	892 (33.7%)	11 (0.4%)	7 (0.3%)
Female (*n* = 1736)	1647 (62.2%)	57 (2.2%)	32 (1.2%)
Affirmed gender			
Male (*n* = 892)	892 (33.7%)		
Female (*n* = 1647)	1647 (62.2%)		
Transgender female (*n* = 10)		10 (0.4%)	
Transgender male (*n* = 58)		58 (2.2%)	
Non-binary (*n* = 18)			18 (0.7%)
Genderqueer (*n* = 21)			21 (0.8%)
Sexual attraction			
Heterosexual (*n* = 2328)	2279 (86.1%)	32 (1.2%)	17 (0.6%)
Bisexual/homosexual (*n* = 150)	119 (4.5%)	18 (0.7%)	13 (0.5%)
Queer/others (*n* = 168)	141 (5.3%)	18 (0.7%)	9 (0.3%)
Ethnic group			
Han (*n* = 1448)	1388 (52.5%)	36 (1.4%)	24 (0.9%)
Other (*n* = 1198)	1151 (43.5%)	32 (1.2%)	15 (0.6%)
Residence			
City (*n* = 550)	531 (20.1%)	12 (0.5%)	7 (0.3%)
Town (*n* = 1375)	1311 (49.5%)	36 (1.4%)	28 (1.1%)
Country (*n* = 721)	697 (26.3%)	20 (0.8%)	4 (0.2%)
Being the only child			
Yes (*n* = 440)	418 (15.8%)	11 (0.4%)	11 (0.4%)
No (*n* = 2206)	2121 (80.2%)	57 (2.2%)	28 (1.1%)
Family economic status			
Rich (*n* = 102)	97 (3.7%)	2 (0.1%)	3 (0.1%)
Moderate (*n* = 1746)	1677 (63.4%)	42 (1.6%)	27 (1.0%)
Poor (*n* = 798)	765 (28.9%)	24 (0.9%)	9 (0.3%)
History of psychiatric disorders			
Yes (*n* = 123)	110 (4.2%)	7 (0.3%)	6 (0.2%)
No (*n* = 2523)	2429 (91.8%)	61 (2.3%)	33 (1.2%)
Psychiatric medication			
Yes (*n* = 42)	40 (1.5%)	1 (0.0%)	1 (0.0%)
No (*n* = 2604)	2499 (94.4%)	67 (2.5%)	38 (1.4%)

Transgender includes binary transgender female and binary transgender male.

Of the total participants, 2539 (96.0%) self-identified as cisgender (male or female), 68 (2.6%) as binary transgender and 39 (1.5%) as non-binary or genderqueer. Sexual attraction differed among all participants, with 2328 (88.0%) self-reporting as heterosexual, 123 (4.6%) as bisexual, 27 (1.0%) as homosexual (lesbian or gay), 109 (4.1%) as queer and 59 (2.2%) as other (e.g. asexual).

### Psychometric properties of the UGDS-GS

We first tested the ceiling and floor effects in the Chinese version of the UGDS-GS.^29^ The total score ranged from 18 to 90. The results showed that 2.6% scored 18 and 0.3% scored 90 (both <15%), indicating that the Chinese version of the UGDS-GS did not demonstrate ceiling or floor effects, which indicated good sensitivity of this instrument.

#### Construct validity

Half of the sample was randomly chosen to conduct EFA. Results of the Kaiser-Meyer-Olkin (KMO) measure of sampling adequacy test and the Bartlett test of sphericity showed that the data was suitable for EFA (KMO = 0.93, *χ^2^* = 13 342.50, d.f. = 153, *P* < 0.001). Using PCA and based on the criterion of Eigenvalues being >1, three factors were exacted, accounting for 61.5% of the total variance.^30^ To be consistent with the original two-factor structure, a fixed two-factor model was performed by using PCA and oblimin rotation, with pairwise deletion of missing data. The exacted two factors explained 55.6% of the total variance. In addition, the original factor names (i.e. dysphoria, gender affirmation) were unchanged in the Chinese version. Dysphoria factors indicated distress about one's physical characteristics, expected behaviours and sense of self in their assigned gender; the gender affirmation factor indicated complete agreement with the benefits of living in the affirmed gender.^18^ Table 2 shows the factor loadings and community of each item. All items loaded on the two factors the same as the original version except for item 10 and 14, which both loaded on gender affirmation factors in the Chinese version but loaded as dysphoria factors in the original version.

**Table 2:** Exploratory factor analysis of the Chinese version of the Utrecht Gender Dysphoria Scale – Gender Spectrum (*N* = 1323)

Items	Factor 1	Factor 2	Communality
13. I feel hopeless if I have to stay in my assigned sex.	0.86		0.75
17. I feel uncomfortable behaving like my assigned sex.	0.85		0.73
15. I feel unhappy because I have the physical characteristics of my assigned sex.	0.85		0.74
12. My life would be meaningless if I would have to live as my assigned sex.	0.82		0.66
18. It would be better not to live, than to live as my assigned sex.	0.79		0.65
16. I hate my birth assigned sex.	0.78		0.64
7. It is uncomfortable to be sexual in my assigned sex.	0.77		0.73
8. Puberty felt like a betrayal.	0.75		0.63
6. I feel unhappy when I have to behave like my assigned sex.	0.73		0.71
9. Physical sexual development was stressful.	0.66		0.46
11. The bodily functions of my assigned sex are distressing for me (i.e. erection, menstruation).	0.64		0.42
2. Every time someone treats me like my assigned sex, I feel hurt.	0.60		0.54
3. It feels good to live as my affirmed gender.		0.87	0.73
4. I always want to be treated like my affirmed gender.		0.86	0.73
1. I prefer to behave like my affirmed gender.		0.75	0.55
5. A life in my affirmed gender is more attractive for me than a life in my assigned sex.		0.56	0.51
10. I wish I had been born as my affirmed gender.		0.53	0.42
14. I feel unhappy when someone misgenders me.		0.34	0.48

Factor 1, dysphoria; factor 2, gender affirmation. The third and fourth columns were factor loadings for each item. Factor loadings <0.3 were omitted.

To further confirm the rationality of the two-factor structure, we performed CFA with the maximum likelihood method. Based on the theoretical framework and model modification indices, we constructed a two-factor model, which showed fair fit (*χ^2^/*d.f. = 9.52, RMSEA = 0.080, CFI = 0.924, TLI = 0.908, SRMR = 0.074). The Chinese version of the UGDS-GS kept the same factor loadings as the original scale, except for the item 5, which was loaded on the dysphoria factor. To test the robustness of the two-factor model, we re-conducted CFA with another sample, and found that the results were robust in these different samples (see Supplementary Material available at https://doi.org/10.1192/bjo.2022.617).

#### Discriminant validity

**Fig. 1:**
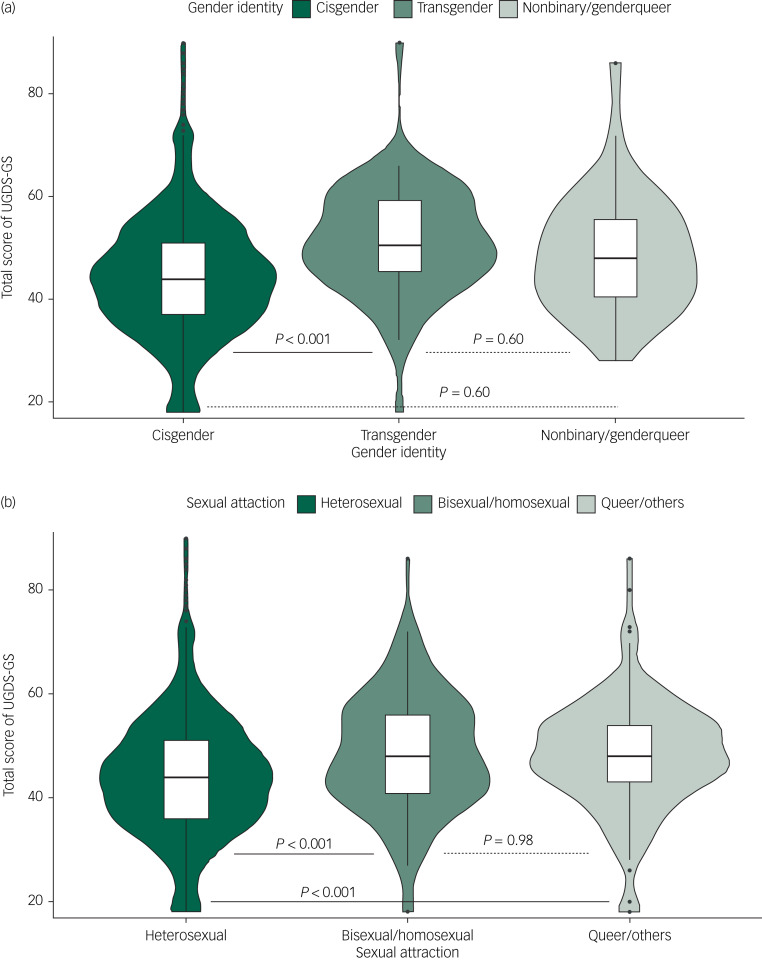
(a) Comparison of the UGDS-GS scores among three gender identity groups (*N* = 2646). (b) Comparison of the UGDS-GS scores among three sexual attraction groups (*N* = 2646). The solid line indicates that the difference between the two groups is significant, and the dashed line indicates that it is non-significant. UGDS-GS, Utrecht Gender Dysphoria Scale – Gender Spectrum.

We verified the discriminant validity of the UGDS-GS for gender identity and sexual attraction. No significant differences were observed between the natal males and natal females (*P* = 1.0), transgender females and transgender males (*P* = 0.52), or non-binary and genderqueer groups (*P* = 0.99); thus, we tested three groups: cisgender, transgender and non-binary/genderqueer. Levene's test of homogeneity of variances showed that the variance was homogeneous (*P* = 0.49), so we used the one-way ANOVA and Scheffe's *post hoc* test to compare the total UGDS-GS scores among the three gender identity subgroups (Fig. 1(a)). Similarly, we compared the total scores among the three sexual attraction subgroups (Fig. 1(b)). Results of ANOVA showed that total score of the UGDS-GS was significantly different among the three gender identity subgroups (*F*(2, 2643) = 14.04, *P* < 0.001). Scheffe's *post hoc* test showed that the transgender group (mean 51.21, s.d. = 10.51) showed significantly higher gender dysphoria scores than the cisgender group (mean 44.28, s.d. = 11.81; *P* < 0.001). No significant differences were found between the non-binary/genderqueer group (mean 48.79, s.d. = 11.48) and the cisgender group (*P* = 0.06), or between the non-binary/genderqueer group and the transgender group (*P* = 0.60). Furthermore, the results showed that there was a significant difference among the three sexual attraction subgroups (*F*(2, 2643) = 17.44, *P* < 0.001). The heterosexual group (mean 44.03, s.d. = 11.84) demonstrated significantly lower gender dysphoria scores than the bisexual/homosexual group (mean 48.33, s.d. = 11.48; *P* < 0.001) and queer/other group (mean 48.04, s.d. = 10.88; *P* < 0.001). No significant differences were found between the bisexual/homosexual group and the queer/other group (*P* = 0.98). On item level, as shown in Supplementary Table 2, the trend of the gender identity group difference for most items was equivalent to the group differences for the total UGDS-GS scores, except for a few items (i.e. items 1, 3, 4, 11, 13, 14, 16 and 18).

#### Reliability

The reliability of the Chinese version of the UGDS-GS was good (Cronbach's *α* = 0.89). The internal consistency and reliability of two subscales were also calculated, with the dysphoria subscale having excellent reliability (Cronbach's *α* = 0.91) and the gender affirmation subscale having good reliability (Cronbach's *α* = 0.83).

#### Criterion validity and convergent validity

As shown in Table 3, results of the Pearson correlation showed that total score of the UGDS-GS was positively associated with anxiety symptoms (*r* = 0.29, *P* < 0.001), depressive symptoms (*r* = 0.31, *P* < 0.001), suicidal ideation (*r* = 0.07, *P* < 0.001) and suicide attempt (*r* = 0.05, *P* < 0.001), indicating good criterion validity of the UGDS-GS. Moreover, the correlation between UGDS-GS and GIDYQ-AA was acceptable (*r* = 0.29, *P* < 0.001), indicating acceptable convergent validity of the UGDS-GS.

**Table 3:** Correlations between total score of the Utrecht Gender Dysphoria Scale – Gender Spectrum and other variables (*N* = 2646)

Variables	Mean	s.d.	1	2	3	4	5	6	7	8
1 UGDS-GS	44.53	11.84	1							
2 Gender affirmation subscale	11.73	2.92	0.35***	1						
3 Dysphoria subscale	32.79	11.14	0.97***	0.11***	1					
4 GIDYQ-AA	44.51	14.14	0.29***	−0.19***	0.36***	1				
5 Anxiety	5.57	4.22	0.29***	0.04*	0.30***	0.24***	1			
6 Depression	6.21	5.11	0.31***	0.02	0.32***	0.28***	0.81***	1		
7 Suicidal ideation	1.29	0.73	0.07***	−0.03	0.09***	0.14***	0.33***	0.38***	1	
8 Suicide attempt	1.14	0.50	0.05**	−0.04	0.07***	0.12***	0.22***	0.24***	0.35***	1
9 Self-harm	1.34	0.83	0.04	−0.03	0.05*	0.12***	0.26***	0.27***	0.37***	0.39***

The gender affirmation subscale and dysphoria subscale are two subscales in the Chinese version of the UGDS-GS. UGDS-GS, Utrecht Gender Dysphoria Scale – Gender Spectrum; GIDYQ-AA, Gender Identity/Gender Dysphoria Questionnaire for Adolescents and Adults.

*P < 0.05; **P < 0.01; ***P < 0.001.

#### Sensitivity and specificity

**Fig. 2:**
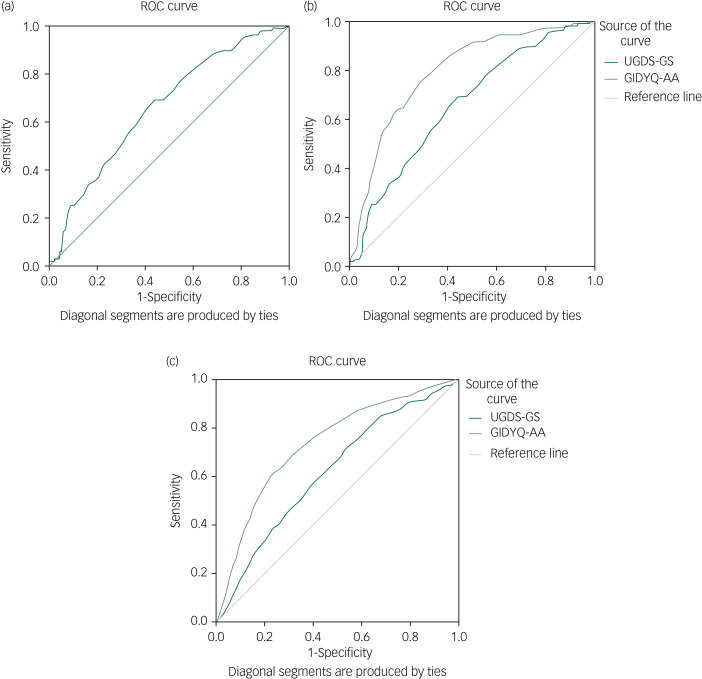
(a) ROC curve of the Chinese version of the UGDS-GS (*N* = 2646; AUC = 0.659, *P* < 0.001). The positive cases include transgender, binary and genderqueer groups. The cut-off score was recommended at 46 (sensitivity 0.69, specificity 0.56). (b) ROC curve of the Chinese version of the UGDS-GS and the GIDYQ-AA in discerning gender identity (*N* = 2646). The larger area under the curve indicates higher power of the scale (AUC_UG_ = 0.659, *P* < 0.001; AUC_GA_ = 0.793, *P* < 0.001). The positive cases include transgender, binary and genderqueer groups. The cut-off score for the UGDS-GS was recommended at 46 (sensitivity 0.69, specificity 0.56); the cut-off for the GIDYQ-AA was recommended at 48 (sensitivity 0.69, specificity 0.56). (c) ROC curve of the Chinese version of the UGDS-GS and the GIDYQ-AA in discerning sexual attraction (*N* = 2646). The larger area under the curve indicates higher power of the scale (AUC_UG_ = 0.617, *P* < 0.001; AUC_GA_ = 0.735, *P* < 0.001). The positive cases include bisexual, homosexual, queer and other diverse individuals. AUC, area under the curve; GIDYQ-AA, Gender Identity/Gender Dysphoria Questionnaire for Adolescents and Adults; ROC, receiver operating characteristic; UGDS-GS, Utrecht Gender Dysphoria Scale – Gender Spectrum.

Figure 2(a) shows the ROC curve of the Chinese version of the UGDS-GS (AUC = 0.66, *P* < 0.001). The optimal cut-off score was 46, with a sensitivity of 0.69 and specificity of 0.56, where the Youden's index reached the maximum (*J =* 0.25).

### Comparison between the UGDS-GS and GIDYQ-AA

The total score of the Chinese version of the GIDYQ-AA ranged from 27 to 135, with no ceiling (0% scored 135) or floor effects (5.3% scored 27). The mean total scores of the UGDS-GS and GIDYQ-AA were 44.53 (s.d. = 11.84) and 44.51 (s.d. = 14.14), respectively. The Cronbach's alpha was 0.89 for the Chinese version of the UGDS-GS and 0.90 for the GIDYQ-AA, which suggested that both scales had good reliability. Using the optimal cut-offs of 46 for the UGDS-GS and 48 for the GIDYQ-AA, there was a significant but poor agreement between the two scales (*κ* = 0.32, *P* < 0.001).

**Fig. 3:**
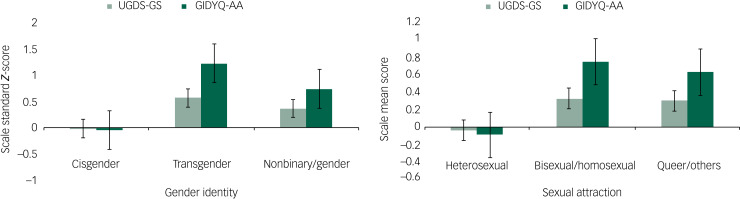
Comparison of the UGDS-GS and GIDYQ-AA among different gender identity and sexual attraction groups (*N* = 2646). The columns show the standard *Z*-score of each scale. Error bars indicate the s.e. GIDYQ-AA, Gender Identity/Gender Dysphoria Questionnaire for Adolescents and Adults; UGDS-GS, Utrecht Gender Dysphoria Scale – Gender Spectrum.

We verified the discriminant validity of the GIDYQ-AA with both gender identity and sexual attraction (Fig. 3). In terms of gender identity, the ANOVA showed that the total GIDYQ-AA scores were significantly different among the three gender identity subgroups (*F*(2, 2643) = 67.43, *P* < 0.001): the transgender group (mean 61.84, s.d. = 17.71) showed significantly higher gender dysphoria than the cisgender group (mean 43.89, s.d. = 13.67; *P* < 0.001), and the non-binary/genderqueer group (mean 54.97, s.d. = 14.79) showed significantly higher dysphoria than the cisgender group (*P* < 0.001), but there was no significant difference between the transgender and non-binary/genderqueer group (*P* = 0.099). It is worth noting that the non-binary/genderqueer group also showed significantly higher dysphoria than the cisgender group (*P* < 0.001) in the GIDYQ-AA, but this was not significant (*P* = 0.060) in the UGDS-GS. As for sexual attraction, the heterosexual group (mean 43.19, s.d. = 13.42) demonstrated significantly lower GIDYQ-AA scores than both the bisexual/homosexual group (mean 55.07, s.d. = 15.18; *P* < 0.001) and the queer/other group (mean 53.39, s.d. = 15.77; *P* < 0.001). No significant difference was observed between the bisexual/homosexual and queer/other group (*P* = 0.704). The results were consistent with that of the UGDS-GS. That is, the total scores of the UGDS-GS were positively associated with that of the GIDYQ-AA (*r* = 0.29, *P* < 0.001) (Table 3). Similar to the UGDS-GS, the GIDYQ-AA score was positively associated with anxiety symptoms (*r* = 0.24, *P* < 0.001), depressive symptoms (*r* = 0.28, *P* < 0.001), suicidal ideation (*r* = 0.14, *P* < 0.001) and attempted suicide (*r* = 0.12, *P* < 0.001).

Figures 2(b) and 2(c) show the ROC curves of the UGDS-GS and GIDYQ-AA in gender identity and sexual attraction, respectively. Comparison of AUC statistics showed that the GIDYQ-AA had better diagnostic power than the UGDS-GS with both gender identity (AUC_GA_ = 0.79, AUC_UG_ = 0.66) and sexual attraction (AUC_GA_ = 0.74, AUC_UG_ = 0.62). The optimum cut-off score of the GIDYQ-AA was 48, based on the maximum of Youden's index (*J =* 0.47), which was consistent with previous studies. On the basis of the optimum cut-offs scores, the GIDYQ-AA (sensitivity 0.76, specificity 0.71) had higher sensitivity and specificity than the UGDS-GS (sensitivity 0.69, specificity 0.56).

## Discussion

This is the first validation study for the UGDS-GS, which also compared the psychometric properties between the GIDYQ-AA and the UGDS-GS. The Chinese version of the UGDS-GS demonstrated good psychometric properties with high internal reliability and good validity. The Chinese version of the UGDS-GS showed a consistent two-factor structure (i.e. dysphoria, gender affirmation) that was the same as the original scale, with slight deviations on the item loadings on factors. Unlike the hypothesis, UGDS-GS did not outperformance the GIDYQ-AA in assessing gender dysphoria in non-binary and queer individuals. Our results showed that GIDYQ-AA was more sensitive for assessing the gender dysphoria differences between non-binary/genderqueer group and cisgender, outperforming the UGDS-GS.

The current study continued the novel contributions of the UGDS-GS in gender dysphoria research, which further expanded knowledge on gender dysphoria in non-binary, queer, LGBTQ and cisgender heterosexual people in a Chinese population. Both the GIDYQ-AA and the UGDS-GS demonstrated good psychometric properties, with the GIDYQ-AA showing relatively better psychometric properties than the UGDS-GS. However, the UGDS-GS has a gender-neutral version for gender dysphoria measurement, which has special application values for groups such as non-binary.

Non-binary individuals identify differently from the traditional female and male binary categories; they may identify with both genders, outside the gender binary or no gender.^31,32^ Although there are approximately a third of transgender individuals who identify as non-binary,^32^ recent research indicates that non-binary individuals experience gender dysphoria in a unique way.^12^ Research highlights that clinical definitions of gender dysphoria primarily centred on gender binary conceptualisation, and gender dysphoria assessments that reflect non-binary experiences, are needed.^12^ From this perspective, the UGDS-GS has historical importance for trying to capture the gender dysphoria experiences of non-binary individuals. The results showed that transgender individuals had significantly higher scores on the UGDS-GS than cisgender individuals; however, no significant difference was found between the cisgender and non-binary/genderqueer groups, and between the transgender and non-binary/genderqueer groups. That is, although we cannot capture the significant difference in gender dysphoria experience in the transgender and non-binary/genderqueer groups, the results showed that the level of gender dysphoria experienced was different in the three groups. Moreover, the UGDS-GS was also suitable for individuals after gender-affirmative care, such as a gender confirmation survey or someone in the process of transitioning (if that is their goal).

In addition, the factor loadings of the original version and the Chinese version also showed slight differences. Item 5 ‘A life in my affirmed gender is more attractive for me than my assigned sex’ loaded in the gender affirmation subscale in the original version, but loaded in the dysphoria subscale in the Chinese version. In addition, the current results also showed that compared with the dysphoria subscale, the gender affirmation subscale had relatively lower scores when compared with the total score. This could be because of the differences in social contexts and cultural environment. In Chinese society, the transgender group is marginalised and faces considerable social discrimination.^17,33^ As a result, being able to live in the affirmed gender could be more important for decreasing gender dysphoria, rather than having a sense of gender affirmation.

A previous study in Finland showed that adolescent boys were more likely to have gender dysphoria than girls.^13^ However, the current study results did not find significant differences between males and females in both the cisgender and transgender groups. Another previous study indicated that adolescents referred for gender dysphoria are more likely to have emotional problems than non-referred individuals.^34^ This study's results were consistent with previous research and showed gender dysphoria was significantly positively associated with anxiety symptoms, depressive symptoms, suicidal ideation and suicide attempt. Research indicates a noticeable difference in the mental health problems of transgender people, which could be a consequence of stigma and minority stress.^1,35,36^ These results showed that when compared with the heterosexual group, the sexual minority groups experienced higher levels of gender dysphoria. This could be because of gendered stereotypes that aim to categorise gender into specific social roles,^37^ and the incongruence with expected social roles in the sexual minority group.

Several limitations in this study need to be noted. First, the participants in the current sample were young, which is not representative for all age groups. We recommend that the UGDS-GS should be further validated in different age groups. Second, marital status was not collected; however, the mean participant age was 19.3 years. The legal age for marriage in China is 22 years for males and 20 years for females. Marriage is not prevalent during the college period in China.^38^ Thus, almost all participants were unmarried in this study and future research should aim to explore marital status. Third, for the sensitivity and specificity of the UGDS-GS, we used the self-reported gender identity rather than a gender dysphoria clinical diagnosis. However, according to Ashley,^1^ a diagnosis of gender dysphoria can pathologize gender dysphoria, and diagnosis should not be clinically required to access transition-related affirmative interventions as gender identities develop naturally, and transgender identities are non-pathological. Furthermore, a screening tool, such as the UGDS-GS, can measure distress, which may be more cost-effective than a diagnosis because there are a limited number of psychiatrists fluent in Chinese (as part of the limited target medical services available), and few medical facilities provide transgender care.^39^ Fourth, we did not measure the onset age of gender dysphoria. Research has indicated that individuals with an early onset of gender dysphoria could experience higher gender dysphoria than individuals with a late onset, because individuals with late onset are usually older and potentially better at coping with distress.^14^ Future studies should investigate the influence of gender dysphoria onset and level of gender dysphoria by using the UGDS-GS, to further explore the UGDS-GS measurement.

In conclusion, the Chinese version of the UGDS-GS demonstrated good psychometric properties, and showed the association between gender dysphoria and mental health problems. This Chinese version of the UGDS-GS provided the first validated gender-neutral assessment of gender dysphoria for use in Chinese populations, which could promote the understanding of gender dysphoria assessment and transgender research in China and in Chinese-speaking populations around the world.

## Supplementary material

For supplementary material accompanying this paper visit http://doi.org/10.1192/bjo.2022.617. click here to view supplementary material
