# Supplementary material for: Utrecht Gender Dysphoria Scale – Gender Spectrum in a Chinese population: scale validation and associations with mental health, self-harm and suicidality
Source: BJPsych Open. 2023 Jan 18;9(1):e17. doi: 10.1192/bjo.2022.617 (PMC9885349; doi:10.1192/bjo.2022.617)
Supplement: Supplementary file 1 [file S2056472422006172sup001.docx]

**Utrecht Gender Dysphoria Scale-Gender Spectrum in Chinese Population: Scale Validation and Associations with Depression, Anxiety, Self-harm and Suicidality**

**Supplemental Materials**

**Re-validation of the Two-Factor Structure of UGDS-GS**

**Method**

***Participants and procedure***

This study was part of a large-scale, cross-sectional study among college students that was conducted online from October 26^th^ to November 18^th^, 2021 in Jilin province, China. All 63 Universities and Colleges in this province were invited to participate in this study. A Quick Response Code (QR Code) linked to the introduction, invitation, and questionnaire was distributed by all the Universities and Colleges by them forwarding the code to students. Eligibility inclusion criteria included: 1) age above 16 years; 2) students studying in Universities or Colleges in Jilin province, China; 3) able to understand the assessment content and Chinese. Jilin University granted ethical approval for this study. Electronic informed consent was obtained online from all participants.

***Measures***

Sociodemographic characteristics included: age, birth-assigned sex, ethnic group, residence, whether the only child in the family, family economic income, and history of psychiatric disorders. Sexual orientation was assessed through sexual identity and sexual attraction. Sexual identity included six categories: male, female, transgender female, transgender male, non-binary, and genderqueer. Sexual attraction was classified into five categories: heterosexual, bisexual, homosexual (lesbian/gay), queer, and others (e.g., asexual). Gender dysphoria was assessed by the UGDS-GS (McGuire et al., 2020).

***Analytic approach***

All statistical analyses were conducted using IBM SPSS 23.0, and Mplus 8.3. Descriptive statistics were generated for each item score and the sociodemographic characteristics. In order to confirm the fitness of the two-factor structure derived from EFA and test the robustness of the two-factor model in China, another Confirmatory Factor Analysis (CFA) using the Jilin sample was performed with Maximun Likelihood (ML) estimates. The goodness of model fit was evaluated by a number of statistics, i.e., chi-squared-degree of freedom ratio (*χ^2^/df*), Root Mean Square Error of Approximation (RMSEA), Comparative Fit Index (CFI), Tucker-Lewis Index (TLI), and Standardized Root Mean Residual (SRMR) (Hu & Bentler, 1998). Acceptable goodness-of-fit model parameters were defined as RMSEA < .08, CFI > .90, TLI > .90, SRMR < .08 (Zhonglin et al., 2004).

**Results**

***Sociodemographic characteristics***

A total of 96,218 participants composed the final sample (see *Table S1*). The age ranged from 15 to 47 years old (*M* = 19.59, *SD* = 1.74). Of all the participants, 90,344 (93.9%) self-identified as cisgender (males or females), 2,675 (2.8%) as binary transgender, and 3,199 (3.3%) as nonbinary or gender queers. Sexual attraction differed among all participants, with 83,721 (87.0%) self-reporting as heterosexual, 6,428 (6.7%) bisexual, 1,331 (1.4%) homosexual (lesbian or gay), 1,633 (1.7%) queer, and 3,105 (3.2%) as others (e.g., asexual).

***Psychometric properties of the UGDS-GS***

The ceiling and floor effects were examined in the Chinese version of UGDS-GS. The total score ranged from 18 to 90. The results showed that 3.3% scored 18 and 0.0% scored 90 (both less than 15%), indicating the Chinese version of the UGDS-GS did not demonstrate ceiling or floor effects, which indicated good sensitivity of this instrument in this sample.

***Construct validity***

To further test the robustness of the two-factor structure, the researchers performed CFA with ML method. As *Figure S1* shows, the two-factor model showed fair fit (*χ^2^/df* = 588.78, RMSEA = 0.078, CFI = 0.929, TLI = 0.917, SRMR = 0.077). The results showed that the structure was consistent with that mentioned in the body of the paper.

**References**

Hu, L., & Bentler, P. M. (1998). Fit indices in covariance structure modeling: Sensitivity to underparameterized model misspecification. *Psychological Methods*, *3*(4), 424-453. <https://doi.org/10.1037/1082-989X.3.4.424>

McGuire, J. K., Berg, D., Catalpa, J. M., Morrow, Q. J., Fish, J. N., Nic Rider, G., Steensma, T., Cohen-Kettenis, P. T., & Spencer, K. (2020). Utrecht Gender Dysphoria Scale-Gender Spectrum (UGDS-GS): construct validity among transgender, nonbinary, and LGBQ samples. *International Journal of Transgender Health*, *21*(2), 194-208.

Zhonglin, W., Kit-Tai, H., & Marsh, H. W. (2004). Structural equation model testing: Cutoff criteria for goodness of fit indices and Chi-square test. *Acta Psychologica Sinica*, *36*(2), 186-194.

**Table S1.** Sociodemographic characteristics of participants (*N* = 96,218)

| Variables | Cisgender  (*N* = 90344) | Transgender  (*N* = 2675) | Nonbinary/genderqueer  (*N* = 3199) | *χ^2^-*test/ANOVA |
| --- | --- | --- | --- | --- |
|  |  | *M* ± *SD / n (%)* |  | *p* value |
| Age (19.59 ± 1.74) | 19.59 ± 1.74 | 19.58 ± 1.80 | 19.62 ± 1.71 | 0.655 |
| Birth-assigned sex |  |  |  |  |
| Male (*N* = 40065) | 38745 (40.3%) | 547 (0.6%) | 773 (0.8%) | < 0.001 |
| Female (*N* = 56153) | 51599 (53.6%) | 2128 (2.2%) | 2426 (2.5%) |  |
| Affirmed gender |  |  |  |  |
| Male (*N* = 38891) | 38891 (40.4%) |  |  |  |
| Female (*N* = 51453) | 51453 (53.5%) |  |  |  |
| Transgender female (*N* = 544) |  | 544 (0.6%) |  |  |
| Transgender male (*N* = 2131) |  | 2131 (2.2%) |  |  |
| Nonbinary/queer (*N* = 3199) |  |  | 3199 (3.3%) |  |
| Sexual attraction |  |  |  | < 0.001 |
| Heterosexual (*N* = 83721) | 80913 (84.1%) | 1438 (1.5%) | 1370 (1.4%) |  |
| Bisexual/homosexual (*N* = 7759) | 5889 (6.1%) | 902 (0.9%) | 968 (1.0%) |  |
| Queer/others (*N* = 4738) | 3542 (3.7%) | 335 (0.3%) | 861 (0.9%) |  |
| Ethnic group |  |  |  | < 0.001 |
| Han (*N* = 86111) | 80954 (84.1%) | 2337 (2.4%) | 2820 (2.9%) |  |
| Others (*N* = 10107) | 9390 (9.8%) | 338 (0.4%) | 379 (0.4%) |  |
| Residence |  |  |  |  |
| City (*N* = 48932) | 45724 (47.5%) | 1400 (1.5%) | 1808 (1.9%) | < 0.001 |
| Town/country (*N* = 47286) | 44620 (46.4%) | 1275 (1.3%) | 1391 (1.4%) |  |
| Being the only child |  |  |  |  |
| Yes (*N* = 45660) | 42844 (44.5%) | 1186 (1.2%) | 1630 (1.7%) | < 0.001 |
| No (*N* = 50558) | 47500 (49.4%) | 1489 (1.5%) | 1569 (1.6%) |  |
| Family income/Month (RMB) |  |  |  | < 0.001 |
| 6000 (*N* = 28624) | 26732 (27.8%) | 912 (0.9%) | 980 (1.0%) |  |
| 6000-14000 (*N* = 31212) | 29310 (30.5%) | 874 (0.9%) | 1028 (1.1%) |  |
| 14000-23000 (*N* = 16061) | 15108 (15.7%) | 443 (0.5%) | 510 (0.5%) |  |
| 23000-36000 (*N* = 9414) | 8889 (9.2%) | 218 (0.2%) | 307 (0.3%) |  |
| 36000-70000 (*N* = 6486) | 6120 (6.4%) | 138 (0.1%) | 228 (0.2%) |  |
| >70000 (*N* = 4421) | 4185 (4.3%) | 90 (0.1%) | 146 (0.2%) |  |
| History of psychiatric disorders |  |  |  | < 0.001 |
| Yes (*N* = 7552) | 6446 (6.7%) | 474 (0.5%) | 632 (0.7%) |  |
| No (*N* = 88666) | 83898 (87.2%) | 2201 (2.3%) | 2567 (2.7%) |  |

*Note*. Transgender includes binary transgender female and transgender male. Comparisons of the continuous variables were conducted using ANOVA and that of the categorical variables were by *χ^2^* test.

**Figure S1.** The two-factor structure of the Chinese version of UGDS-GS (*N* = 96218)


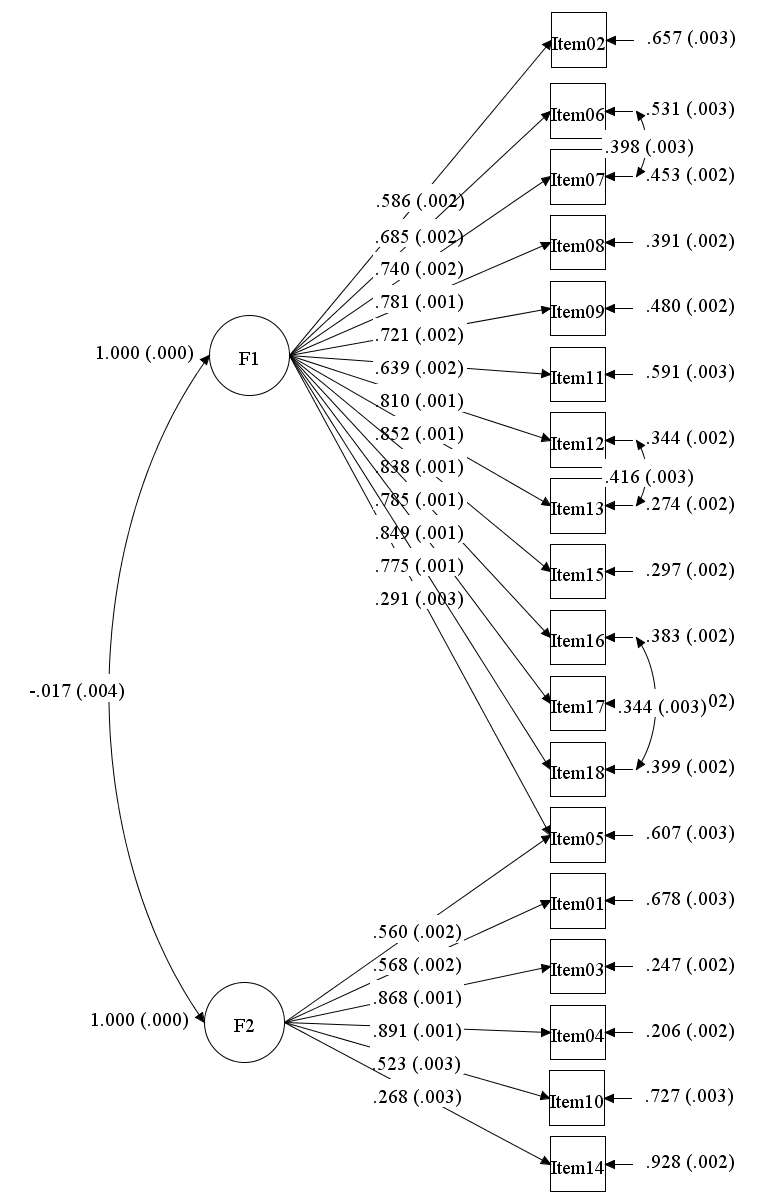


*Note*. Factor 1: Dysphoria; Factor 2: Gender affirmation.
